# Phytochemical-Mediated Biosynthesis of Silver Nanoparticles from *Strobilanthes glutinosus:* Exploring Biological Applications

**DOI:** 10.3390/mi14071372

**Published:** 2023-07-04

**Authors:** Rabia Javed, Shumaila Ijaz, Hajra Hameed, Moona Nazish, Muhammad Shakeeb Sharif, Afshan Afreen, Khaloud Mohammed Alarjani, Mohamed S. Elshikh, Saadia Mehboob, Sarah Abdul Razak, Abdul Waheed, Rashid Ahmed, Muhammad Tariq

**Affiliations:** 1 Department of Biotechnology, Mirpur University of Science and Technology, Mirpur 10250, Pakistanhajrahameedmughal@gmail.com (H.H.); mshakeebsharif@gmail.com (M.S.S.); saadia.mehboob@must.edu.pk (S.M.); 2 Department of Plant Sciences, Quaid-i-Azam University, Islamabad 45320, Pakistan; 3 Department of Botany, Rawalpindi Women University, Rawalpindi 46300, Pakistan; 4 Department of Botany and Microbiology, College of Science, King Saud University, Riyadh 11451, Saudi Arabia; kalarjani@ksu.edu.sa (K.M.A.); melshikh@ksu.edu.sa (M.S.E.); 5 Institute of Biological Sciences, Faculty of Science, Universiti Malaya, Kuala Lumpur 50603, Malaysia; sarahrazak@um.edu.my; 6 Agricultural Genomics Institute at Shenzhen, Chinese Academy of Agricultural Sciences, Shenzhen 518120, China; 7 Nick Holonyak Jr. Micro and Nanotechnology Laboratory, University of Illinois at Urbana Champaign, Urbana, IL 61801, USA; hashmi133@yahoo.com

**Keywords:** *Strobilanthes glutinosus*, silver nanoparticles, antioxidant, antibacterial, phytochemical

## Abstract

The application of green synthesis for silver nanoparticles in nanomedicine has experienced significant growth. *Strobilanthes glutinosus*, a plant primarily located in the Himalayas, remains largely unexplored. Considering the biomedical value of *S. glutinosus*, phytochemicals from this plant were used for the biosynthesis of silver nanoparticles. Silver nanoparticles were synthesized from aqueous extract of root and leaves of *Strobilanthes glutinosus*. The synthesized silver nanoparticles were characterized using UV–Vis spectrophotometry, Fourier-transform infrared spectroscopy, transmission electron microscopy, and X-ray diffraction. Total phenolic and flavonoid contents of plants were determined and compared with nanoparticles. The biomedical efficacy of plant extracts and silver nanoparticles was assessed using antioxidant and antibacterial assays. The UV–Vis spectra of leaf- and root-extract-mediated AgNPs showed characteristic peaks at 428 nm and 429 nm, respectively. TEM images revealed the polycrystalline and spherical shapes of leaf- and root-extract-mediated AgNPs with size ranges of 15–60 nm and 20–52 nm, respectively. FTIR findings shown the involvement of phytochemicals of root and leaf extracts in the reduction of silver ions into silver nanoparticles. The crystalline face-centered cubic structure of nanoparticles is depicted by the XRD spectra of leaf and root AgNPs. The plant has an ample amount of total phenolic content (TPC) and total flavonoid content (TFC), which enhance the scavenging activity of plant samples and their respective AgNPs. Leaf and root AgNPs have also shown good antibacterial activity, which may enhance the medicinal value of AgNPs.

## 1. Introduction

Nanotechnology is a rapidly growing field with diverse applications in various disciplines such as health, drug delivery, cosmetics and environment. It also shows great promise for the detection and treatment of various human disorders [1,2]. Metal nanoparticles (MNPs) have garnered attention due to their biological features, such as enzyme blocking, as well as antibacterial, antifungal, anticancer, and anti-leishmaniasis properties [3]. Moreover, MNPs have applications in diverse sectors such as food, cosmetics, electronics, agriculture, catalysis, drug delivery, and imaging [4]. Silver nanoparticles (AgNPs) have broad applications in nanoscience and nano-biotechnology [5,6]. These nanoparticles possess distinctive chemical and structural characteristics which differentiate them from their bulk counterparts [7,8]. The cellular uptake of nanomaterials is influenced by their shape, with nanorods exhibiting the highest uptake, followed by nanospheres, nanocylinders, and nanocubes [9]. In the pursuit of safer and more sustainable synthesis of MNPs, green methods that employ plants and other natural products have become significantly prominent in recent years [10]. Moreover, green synthesis utilizes phytochemicals instead of hazardous chemicals to reduce and stabilize metal ions during MNPs, resulting in biocompatible and eco-friendly nanomaterials [11,12,13]. Phytochemicals present in plant extracts play a crucial role in reduction processes and impart significant medicinal and biological activities to the nanoparticles [14]. AgNPs synthesized through green methods exhibit antimicrobial potential, combatting multiple drug resistance, and they have applications in cancer theranostics [15]. Strobilanthes is the second-largest genus of family Acanthaceae in Asia, with about 400 species [16]. It is reported that several species of the genus *Strobilanthes glutinosus* have pharmacological potential via their antioxidant, antibacterial and antidiabetic properties [17].

This study focuses on the green synthesis of AgNPs using leaves (leaf AgNPs) and roots (root AgNPs) of *Strobilanthes glutinosus,* followed by a comparison of their phytochemicals and biomedical applications. Leaf AgNPs and root AgNPs were characterized using UV–visible spectroscopy, FTIR, TEM, and XRD.

## 2. Results

### 2.1. Optical Observations

**Figure 1:**
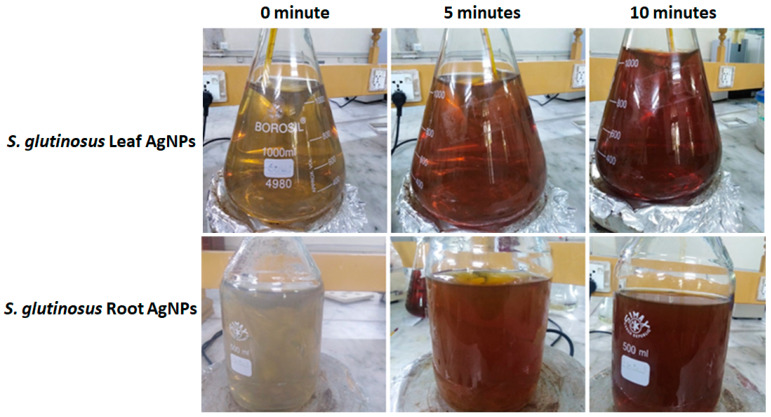
Optical changes observed during the formation of leaf AgNPs (upper set) and root AgNPs (lower set) at 0, 5, and 10 min of mixing.

*When Strobilanthes glutinosus* was used to prepare silver nanoparticles at 70 °C, a color change of the reaction mixture from greenish brown to dark brown was observed, which is a visual indication of silver nanoparticle synthesis from the leaves of *Strobilanthes glutinosus* (as shown in Figure 1). Root Ag NPs were fabricated; with frequent color changes from light yellow to grayish yellow and finally dark brown (Figure 1). This color change is due to the optical properties of silver nanoparticles. The color changes during the synthesis process are the first indication of the synthesis of nanoparticles, as observed in many previous studies, such as deep yellow to dark brown [18], greenish yellow to brown, greenish to dark brown [19], and light yellow to brown [20].

### 2.2. UV–Vis Analysis

**Figure 2:**
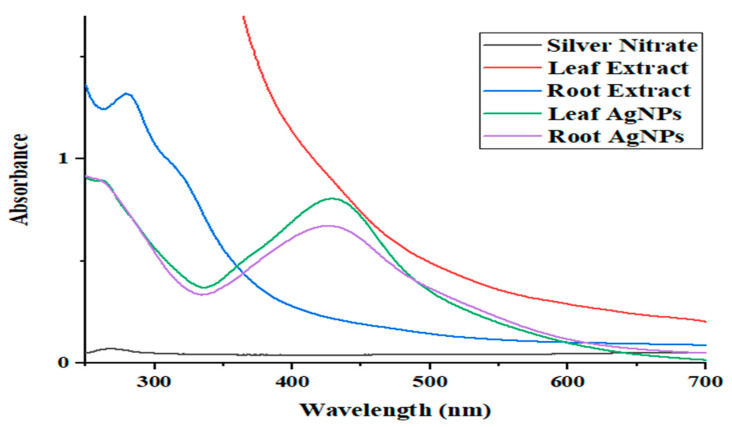
UV–Vis spectra of root AgNPs and leaf AgNPs.

UV–Vis spectroscopy is a technique used to examine the formation of nanomaterials. The peak of silver nanoparticles produced from aqueous leaf extract of *Strobilanthes glutinosus* (leaf AgNPs) was shown at ~429 nm, while the peak of silver nanoparticles produced from aqueous root extract (root AgNPs) was shown at ~428 nm due to surface plasmon resonance (Figure 2). In many previous studies, maximum absorbance peaks were recorded at 417 nm, 430 nm, and 428 nm for silver nanoparticles from the plants *Ajuga bracteosa* [21], *Pedalium murex* [22], and *Erythrina suberosa* [23], respectively. In another study conducted by Salayová and colleagues, silver nanoparticles had absorbance peaks at 421 nm, 422 nm, and 426 nm from the plants *Berberis vulgaris*, *Capsella bursa-pastoris*, and *Origanum vulgare* [24]. Similar results are also reported by Noukelag et al., 2020 [25].

### 2.3. Transmission Electron Microscopy

**Figure 3:**
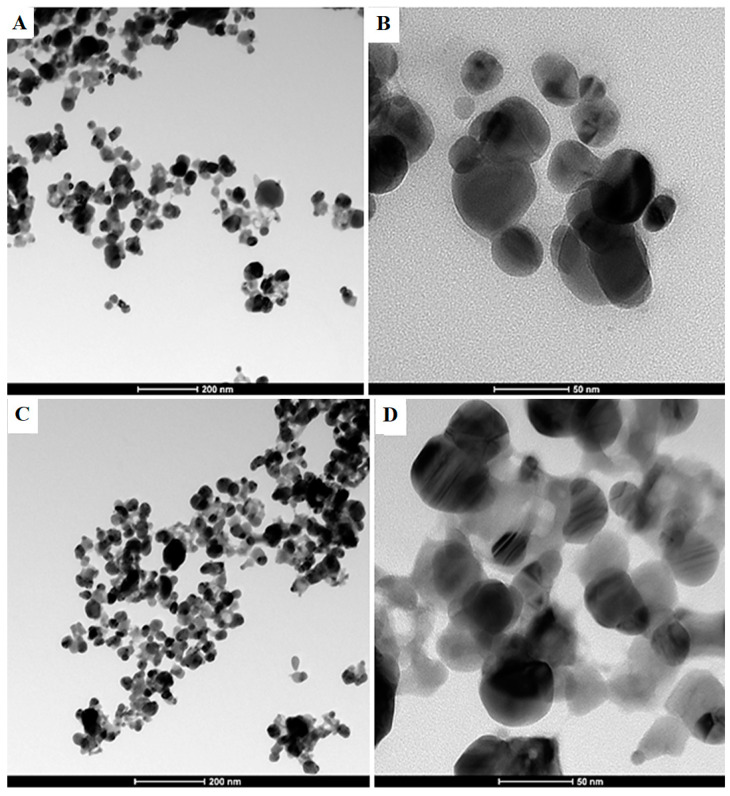
TEM images of leaf AgNPs (**A**,**B**) and root AgNPs (**C**,**D**) at two different magnifications.

Transmission electron microscopy (TEM) depicts the shape, size, crystallinity, and morphology of silver nanoparticles. The TEM images revealed that the silver nanoparticles (both leaf AgNPs and root AgNPs) are polycrystalline and spherical in shape. The sizes of nanoparticles were measured using image J software. The size of leaf AgNPs range from 15–60 nm with average size of 32 ± 12 nm (Figure 3), while root AgNPs range from 20–52 nm with average size of 35 ± 8 nm (Figure 3). In a study conducted by Syed et al. on the synthesis of silver nanoparticles, particles ranging in size from 5 to 50 nm were obtained [26]. In a review article, the sizes of silver nanoparticles produced from various green sources were reported, such as silver nanoparticles produced from coffee, gelatin, glucose, tea, olive extract (1 mL), olive extract (5 mL), *Leptadenia reticulate, Elaeagnus latifolia*, and *Chrysanthemum indicum* L., which were 60 nm, 3.68 nm, 5.28 nm, 60 nm, 30 nm, 15 nm, 50–70 nm, 30–50 nm, and 38–72 nm in size, respectively [27]. In another study, 16 nm AgNPs were produced from *Ficus benghalensis* leaf extract [28]. 

### 2.4. FTIR Analysis

**Figure 4:**
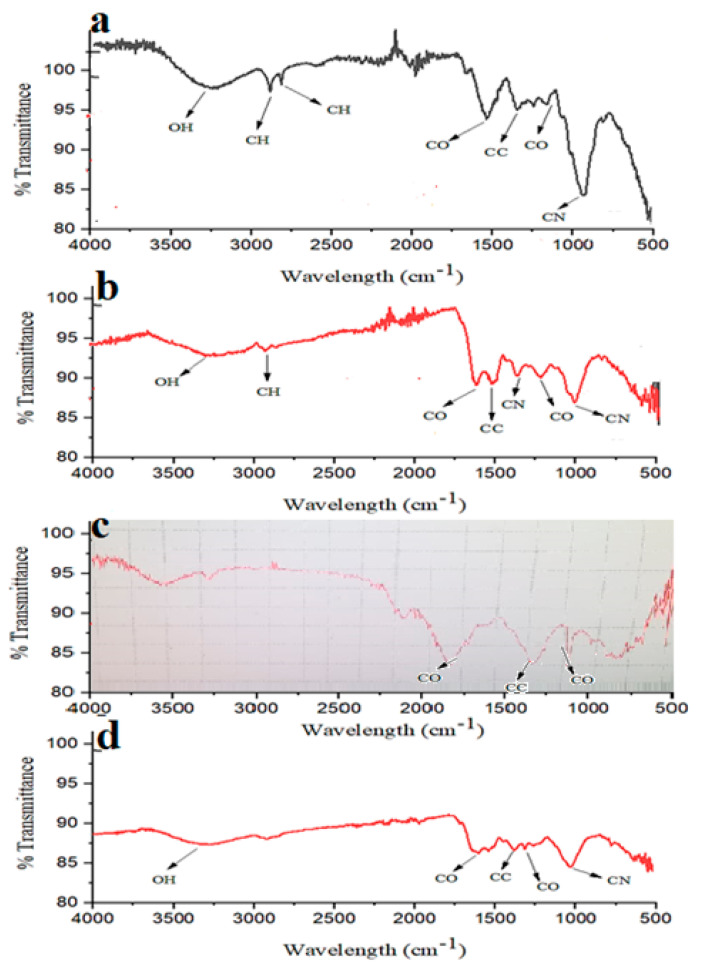
FTIR spectra of leaf extract (**a**), leaf AgNPs (**b**), root extract (**c**), and AgNPs (**d**).

FTIR is carried out to identify the functional groups of biomolecules that are linked to silver nanoparticles. The peaks of pure leaf extract, pure root extract, root AgNPs, and leaf AgNPs on FTIR spectra show slight differences in comparison to each other, as shown in Figure 4. In pure leaf extract, the peaks at 2920 cm^−1^ and 2845 cm^−1^, represent C-H stretching, and similar peaks were also reported in a study by Rajakumar et al. at 1631 cm^−1^, 2922 cm^−1^, and 2853 cm^−1^ [29]. Devaraj and colleagues reported the peaks of silver nanoparticles in their study at 2927 cm^−1^, 1631 cm^−1^, and 1383 cm^−1^, which closely resemble our peaks for leaf AgNPs at 2907 cm^−1^, 1633 cm^−1^, and 1384 cm^−1^, depicting C-H or N-H, C=O, and CN, respectively [30]. Jyoti et al. prepared silver nanoparticles from Urtica dioica leaves, their FTIR spectra showed some peaks at 2921 cm^−1^, 1631 cm^−1^, 1377 cm^−1^, 1240 cm^−1^, and 1043 cm^−1^, which are similar to our leaf AgNPs peaks at 2907 cm^−1^, 1633 cm^−1^, 1384 cm^−1^, 1240 cm^−1^, and 1028 cm^−1^ [31]. In another study, the reported peaks of silver nanoparticles were 3271 cm^−1^, 1633 cm^−1^, 1397 cm^−1^, and 1081 cm^−1^, which are close to our peaks for leaf AgNPs at 3257 cm^−1^, 1633 cm^−1^, 1384 cm^−1^, and 1028 cm^−1^ [32]. In the FTIR spectrum of the pure root samples, a peak at 1617 cm^−1^ was observed, which is closely related to 1636 cm^−1^ [33] and 1631 cm^−1^ [29]. Devaraj reported peaks at 1629 cm^−1^, 1041 cm^−1^, and 833 cm^−1^, which relate to the peaks for our root samples of *Strobilanthes glutinosus* at 1617 cm^−1^, 1027 cm^−1^, and 875 cm^−1^, showing C=O (carbonyl) stretching, C-N (amine) stretching, and C=CH2 stretching [30]. The silver nanoparticles prepared by Jyoti and colleagues had peaks at 1631 cm^−1^, 1377 cm^−1^, and 1043 cm^−1^, which closely resemble our root AgNPs peaks at 1617 cm^−1^, 1386 cm^−1^, and 1033 cm^−1^ [31]. In a study by Dixit et al., peaks found at 1633 cm^−1^, 1397 cm^−1^, and 1081 cm^−1^ resemble our peaks of root AgNPs at 1617 cm^−1^, 1386 cm^−1^, and 1033 cm^−1^ [32]. Devanesan and AlSalhi reported FTIR absorption peaks of silver nanoparticles at 1641 cm^−1^ and 1066 cm^−1^, which are related to peaks at 1617 cm^−1^, and 1033 cm^−1^ for root AgNPs [34]. A study conducted by Settu et al. reports FTIR absorption peaks of silver nanoparticles synthesized using Hydnocarpus alpina aqueous extracts at 3390 cm^−1^, and 1320 cm^−1^, which are close to our peaks of root AgNPs found at 3338 cm^−1^, and 1313 cm^−1^, showing O-H stretching of phenols or water and C=O stretching of carbonyl compounds [35].

### 2.5. X-ray Diffraction Analysis

**Figure 5:**
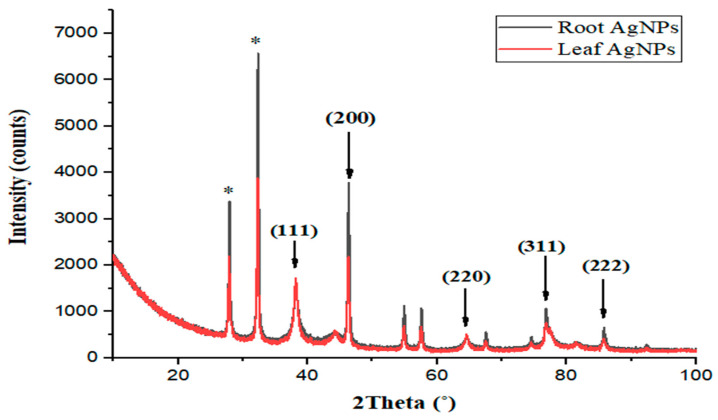
XRD spectra of root and leaf AgNPs.

The XRD analysis of silver nanoparticles synthesized from *Strobilanthes glutinosus* roots and leaves revealed nine peaks at 27.91°, 32.44°, 38.21°, 46.23°, 54.79°, 57.58°, 64.39°, 76.97°, and 85.69°. The high intensity of peaks represents the crystalline structure of nanoparticles (Figure 5). According to the online database, JCPDS, XRD data shown that root and leaf AgNPs have face-centered cubic structures. The XRD absorption peaks at 38.21°, 46.23°, 64.39°, 76.97°, and 85.69° correspond to the (111), (200), (220), (311), and (222) planes of silver, respectively, similar to those reported in a study by Majeed et al. at 38.1°, 44.09°, 64.36°, 77.29°, and 81.3° [36]. In a study conducted by Sulaiman et al. on the synthesis of silver nanoparticles by *Aspergillus flavus*, XRD peaks were obtained at 45.0°, 65.45°, and 78.6°, which are in close similarity to our peaks at 46.23°, 64.39°, and 76.97° [37]. Vanaja and Annadurai carried out a study on the synthesis of silver nanoparticles by using leaf extracts and recorded XRD peaks at 38.19°, 44.18°, 67.44°, 77.70°, which are quite close to our peaks of root AgNPs 39 and leaf AgNPs at 38.21°, 46.23°, 64.39°, and 76.97°, corresponding to the (111), (200), (220), and (311) planes of silver [38]. The phytochemicals present in plant extracts were absorbed on the surface of AgNPs, which is also evident from FTIR analysis, and these findings are supported by several similar studies [39,40,41,42,43,44,45,46,47,48].

### 2.6. Antibacterial Activity

**Figure 6:**
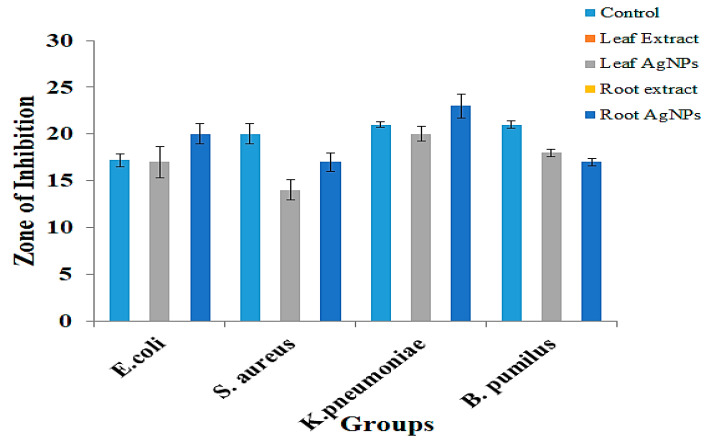
Antimicrobial activities of positive control, leaf extract, leaf AgNPs, root extract, and root AgNPs against *E. coli*, *S. aureus*, *K. pneumoniae*, and *B. pumilus*.

The anti-bacterial activity of silver nanoparticles was evaluated using the well-diffusion method on two Gram-positive bacterial strains, *Staphylococcus aureus* and *Bacillus pumilus,* and two Gram-negative bacterial strains, *Klebsiella pneumoniae* and *Escherichia coli.* Rifampicin was used as a positive control. These extracts did not have any antibacterial activity at all, while leaf AgNPs and root AgNPs showed remarkable results in comparison to positive controls (Figure 6). The average of three replicates of the diameters of zones of inhibition were measured in millimeters. The aqueous root extract and aqueous leaf extract of *Strobilanthes glutinosus* had no activity against any of the bacterial species. In a study conducted by Ajaib and colleagues, it was also reported that the plant extracts of *Strobilanthes glutinosus* have remarkable antibacterial activity in organic solvents like methanol, petroleum ether, and chloroform, but in the case of aqueous extracts, no antibacterial activity was found [17]. In many studies, similar antibacterial activity of silver nanoparticles has been reported against these strains of bacteria. In two different studies conducted by Diana Gabrio and Laura Carson, the silver nanoparticles prepared from *Lysiloma acapulcensis* exhibited zones of inhibition of 18 ± 13 mm against *E. coli* and 16 ± 10 mm against *S. aureus* [49], and the silver nanoparticles prepared from Phyla dulcis exhibited zones of inhibition of 12 mm against both *E. coli* and *S. aureus* [50]. Violeta Morales-Lozoya conducted a study to prepare silver nanoparticles from the extracts of different parts of *Morinda citrifolia*, such as leaf, fruit, and dried seeds, and compared their activities against *E. coli* and *S. aureus*. Zones of inhibition were observed at 18.13 mm, 9.81 mm, and 20.45 mm against *E. coli* and 14.06 mm, 10.63 mm, and 15.10 mm against *S. aureus* from the silver nanoparticles prepared from the fruit extract, leaf extract, and dried seed extract of *Morinda citrifolia*, respectively [51]. A study conducted by Masoud Hussein and colleagues reveals that silver nanoparticles synthesized from onion and ginger extracts against *K. pneumoniae* have zones of inhibition with diameters of 8.33 ± 0.33 mm and 10.33 ± 0.33 mm, respectively [52]. Another study proclaims that silver nanoparticles with a size range of 20 nm to 70 nm have a zone of inhibition of 22 mm diameter against K. pneumoniae [53]. Silver nanoparticles against various strains of *K. pneumoniae* (MF953599 and 40 MF95353600) have similar results as in the present study with a 500 µg/L concentration and the diameter of the zone of inhibition being 34 ± 1 mm and 37 ± 0.5 mm, respectively [54].

### 2.7. Phytochemical Analysis

The phytochemicals are bioactive nutrient plant materials with antioxidant properties that help prevent many chronic diseases and oxidative damage. Phytochemical molecules include carotenoids, phytosterols, limonoids, polyphenols, glucosinolates, phytoestrogens, terpenoids, fibers, polysaccharides, saponins, etc. A phytochemical analysis (total phenolic content, total flavonoid content) of *Strobilanthes glutinosus* and its nanoparticles was performed to evaluate the medicinal and nutritional potential of nanoparticles synthesized from *Strobilanthes glutinosus* extract. 

#### 2.7.1. Total Phenolic Content

The total phenolic contents of the leaf and root parts of *Strobilanthes glutinosus* and their nanoparticles (leaf AgNPs and root AgNPs) were assessed using the calibration curve of gallic acid. The total phenolic contents present in methanolic leaf and root extracts are 8 ± 0.02 mgGAE/g and 1 ± 0.01 mgGAE/g. The methanolic suspensions of leaf AgNPs and root AgNPs have total phenolic contents of 21 ± 0.02 mgGAE/g and 26 ± 0.04 mgGAE/g (Table 1). The calibration curve for gallic acid was obtained with R2 = 0.9701. A study conducted by Prabha and colleagues reports the amount of total phenolic contents as 1.423 mgGAE/g, which is very close to our results for root extract, which are 1 ± 0.01 mgGAE/g [55]. Geethalakshami reported that the total phenolic content of *Sphaeranthus amaranthoides* is 2.15 ± 0.26 mg/g dry weight, which is similar to our results for root and leaf extracts [56]. Malik and colleagues described the total phenolic content of *Arisaema jacquemontii* Blume as 45.17 ± 1.70 mgGAE/g, which is in accordance with our results for root and leaf AgNPs: 26 ± 0.04 mgGAE/g and 21 ± 0.02 mgGAE/g, respectively [57].

#### 2.7.2. Total Flavonoid Content

The calibration curve of rutin was used to evaluate the total flavonoid contents of root and leaf AgNPs. The determination of total flavonoid contents was carried out via the formation of a flavonoid–aluminum complex, where rutin was used as a standard to establish a calibration curve. The total flavonoid content in methanolic leaf and root extracts is 16.9 ± 0.02 mgRE/g and 15.8 ± 0.04 mgRE/g; while the total flavonoid amount that went into the silver nanoparticles is 482.6 ± 0.02 mgRE/g and 504.2 ± 0.04 mgRE/g in leaf AgNPs and root AgNPs (Table 2). A study by Aryal has reported that *Solanum nigrum*, and *Digera muricata* have shown similar amounts of total flavonoid content, i.e., 16.42 ± 0.39 mgQE/g and 18.00 ± 0.68 mgQE/g, respectively [58], which is very close to our results. The results of total flavonoid content and total phenolic contents show that the plant *Strobilanthes glutinosus* and the nanoparticles synthesized from it have high phenolic and flavonoid contents.

### 2.8. Antioxidant Activity

**Figure 7:**
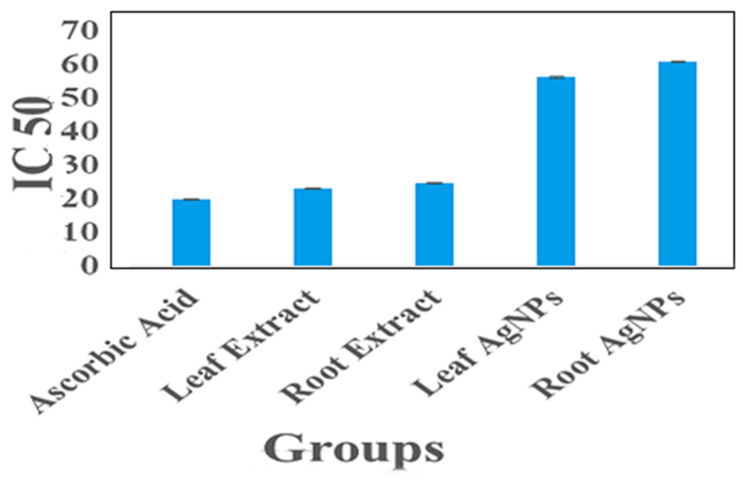
IC_50_ values of ascorbic acid, methanolic extracts of leaf and root samples, leaf AgNPs, and root AgNPs.

**Figure 8:**
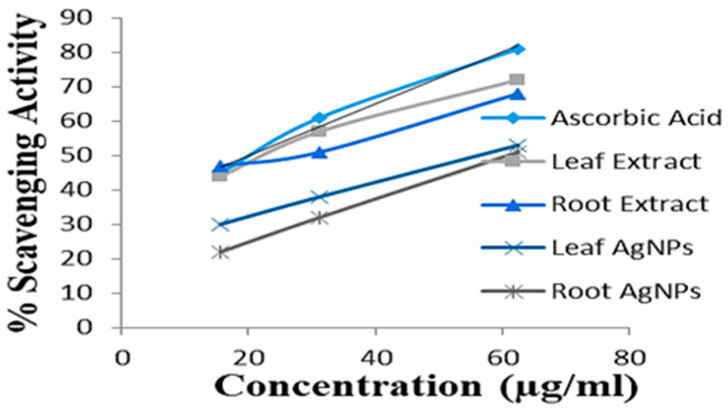
DPPH scavenging activity of ascorbic acid, methanolic extracts of leaf and root samples, leaf and root AgNPs.

The antioxidant activity of silver nanoparticles (synthesized from roots and leaves) was compared with plant samples (root and leaf extracts) via DPPH scavenging activity. The IC_50_ values have shown good antioxidant potential in plant samples and biofabricated silver nanoparticles. The methanolic extracts of plants (roots and leaves) show good antioxidant potential, while the antioxidant potential of leaf AgNPs and root AgNPs was good but less in comparison to the plant samples. The IC_50_ value of ascorbic acid is 20 µg/mL; leaf extract is 23.2 µg/mL, root extract is 24.9 µg/mL; leaf AgNPs are 56.2 µg/mL, and root AgNPs are 60.8 µg/mL. The IC_50_ values of samples and scavenging activity are graphically presented in Figure 7 and Figure 8. The antioxidant activity of silver nanoparticles was observed in comparison to plant (root and leaf) extracts by a most commonly used methods, the DPPH scavenging assay. A lower IC_50_ value depicts high antioxidant activity; the IC_50_ values of ascorbic acid, leaf extract, root extract, leaf AgNPs, and root AgNPs were 20 µg/mL, 41 23 µg/mL, 23.9 µg/mL, 56 µg/mL, and 60.8 µg/mL, respectively, showing that the extracts had better scavenging activity compared to silver nanoparticles. In 2019, a study was conducted by Ahn and colleagues on thirty Chinese plants and silver nanoparticles derived from their extracts to examine their antioxidant, cytotoxic, apoptotic, and wound healing properties. Among those thirty plants, seven (*Cratoxylum formosum*, *Phoebe lanceolata*, *Scurrula parasitica*, *Ceratostigma minus*, *Mucuna birdwoodiana*, *Myrsine africana*, and *Lindera strychnifolia*) had higher antioxidant properties as compared to their green synthesized silver nanoparticles [59]. Silver nanoparticles of similar antioxidant potential as ours were also reported by Wang (IC_50_ = 65 µg/mL) [60] and by Netala (IC_50_ = 63.3 µg/mL) [61].

## 3. Materials and Methods

### 3.1. Green Synthesis of AgNPs from Leaf Extract

Firstly, 1 g of dried leaf powder of *Strobilanthus glutinous* was added to 250 mL of distilled water to prepare leaf extract at 70 °C; it was constantly stirred for 20–25 min and filtered. Then, 750 mL of 1 mM silver nitrate solution were prepared. Next, 245 mL of leaf extract was added to a silver nitrate solution at 70 °C, and an instant color change was observed, indicating the synthesis of silver nanoparticles. The reaction mixture was continuously stirred for 1 h to complete the reaction.

### 3.2. Green Synthesis of AgNPs from Root Extract

For the preparation of AgNPs from root extract, the dried root powder was pre-soaked for 3–7 days. The soaked root powder was boiled for 25–30 min and filtered. The root extract and 1 mM silver nitrate solutions were mixed slowly at 70 °C at 1:3, and the instant color changes depicted the synthesis of nanoparticles, as shown in Figure 1.

### 3.3. Antibacterial Activity of Strobilanthes Glutinous Synthesized AgNPs

The antibacterial study was examined using the well-diffusion method, LB Agar media was poured into the Petri plates, and when the agar media solidified, streaking of the bacterial culture was performed. For digging wells, a well borer was used, and the sample was loaded into the wells. The Petri plates were sealed with parafilm and placed in an incubator at 37 °C for 24 h [61]. The study was conducted against two Gram-positive strains (*S. aureus* and *B. pumilus*) and two Gram-negative strains (*E. coli* and *K. pneumoniae*).

### 3.4. Antioxidant Activity of Strobilanthes Glutinous Synthesized AgNPs

The antioxidant activity of root AgNPs and leaf AgNPs was evaluated using the DPPH scavenging method. The free-radical scavenging activity of silver nanoparticles and plants (root and leaf) was assessed. The samples of plant, ascorbic acid, and silver nanoparticles with concentrations ranging from 50 to 250 µg/mL were dissolved in methanol. Ascorbic acid was used as a control. Each of these solutions was individually added to 1 mL of 0.2 mM DPPH and incubated in the dark for 30 min at room temperature [21]. Absorbance was recorded at 517 nm, and the scavenging ability of each sample was estimated with the following equation:

% Inhibition = Abs. control − Abs. sample/Abs. control × 100(1)

### 3.5. Determination of Total Phenolic Content

The total phenolic content (TPC) of plant extract (i.e., leaf and root separately) and silver nanoparticles (leaf AgNPs and root AgNPs) was determined using the Folin–Ciocalteu reagent method. The phenolic content was determined by the gallic acid calibration curve with its concentrations of 31.62, 62.5, 125, 250, and 500 µg/mL in methanol. Then, 0.5 mL of sample was added to 2.5 mL of 10% FC reagent and 2.5 mL of 7.5% sodium carbonate. The reaction ice was incubated in the dark at room temperature for 30 min, and three consistent readings were taken. The absorbance was measured at 765 nm. The reaction was performed in triplicate. The TPC was calculated using the following formula:

TPC = (XxV)/m; or(2)

(3) $$\mathrm{TPC}=\frac{Gallic\;acid\;concentration\;\left(\frac{mg}{mL}\right)\times Extract\;volume(mL)}{Weight\;of\;tissue\;extract\;(g)}$$

### 3.6. Determination of Total Flavonoid Content

The total flavonoid content (TFC) was determined using a spectrophotometric method based on the formation of the flavonoid–AlCl_3_ complex. Rutin was used as a standard solution to obtain the calibration curve. The methanolic solution of 0.5 mL of sample was added to 0.5 mL of 10% AlCl_3_ solution and 0.75 mL of 5% sodium acetate solution. The reaction mixture was incubated in the dark at room temperature for 2.5 h, and the absorbance was recorded at 440 nm. Three concordance readings were taken.

## 4. Conclusions

Herbal medicines are widely accepted in the medical sector due to their various advantages for human health and their low risk of adverse consequences. According to the current outcomes of phytochemical and antioxidant studies of the samples, *Strobilanthes glutinosis* botanical extracts and metallic nanoparticles may be one of the most therapeutic agents for treating diseases brought on by rising levels of oxidative stress. The rich amounts of phenolics and flavonoids present in both root-based and leaf-based Ag NPs enhanced the antioxidant and antibacterial activities of metallic nanoparticles. The bio fabricated Ag NPs proved to be an effective agent against various types of both Gram-positive and Gram-negative bacterial strains. The green fabrication of Ag NPs may prove a fast, cost-effective, and appropriate alternative to synthetic antibiotics against various multidrug-resistant bacteria.

## Figures and Tables

**Table 1:** Total phenolic contents of methanolic extracts of leaf and root, and methanolic suspensions of leaf AgNPs and root AgNPs.

Groups	Linear Regression Equation	Mean Absorbance of Plant Extract Solution (Y)	Concentration of GAE in Plant Sample (X)	Total Contents Calculated from Equation = (X × V)/m
TPC of leaf extract	Y= 0.0004x + 0.0913	0.842 ± 0.02	1.877	8
TPC of root extract	Y= 0.0004x + 0.0913	0.311 ± 0.01	0.5	1
TPC of Leaf AgNPs	Y= 0.0004x + 0.0913	0.377 ± 0.02	1.78	21
TPC of Root ANPs	Y= 0.0004x + 0.0913	0.335 ± 0.04	0.6	26

**Table 2:** Total flavonoid contents of methanolic extracts of leaf and root, and methanolic suspensions of leaf AgNPs and root AgNPs.

Groups	Linear Regression Equation	Mean Absorbance of Plant Extract Solution (Y)	Concentration of GAE in Plant Sample (X)	Total Contents Calculated from Equation = (X × V)/m
TFC of leaf extract	Y = 0.0032x + 0.0575	1.41 ± 0.02	422.65	16.906
TFC of root extract	Y = 0.0032x + 0.0575	1.96 ± 0.04	594.5	15.84
TFC of leaf AgNPs	Y = 0.0032x + 0.0575	1.63 ± 0.02	482.6	482.6
TFC of root ANPs	Y = 0.0032x + 0.0575	1.67 ± 0.04	504.2	504.2

## Data Availability

We declared that the materials described in the manuscript, including all relevant raw data, will be freely available to any scientist wishing to use them for noncommercial purposes, without breaching participant confidentiality.
